# Supplementary figures and images for: Tubular epithelial progenitors are excreted in urine during recovery from severe acute kidney injury and are able to expand and differentiate in vitro
Source: PeerJ. 2022 Oct 20;10:e14110. doi: 10.7717/peerj.14110 (PMC9588302; doi:10.7717/peerj.14110)

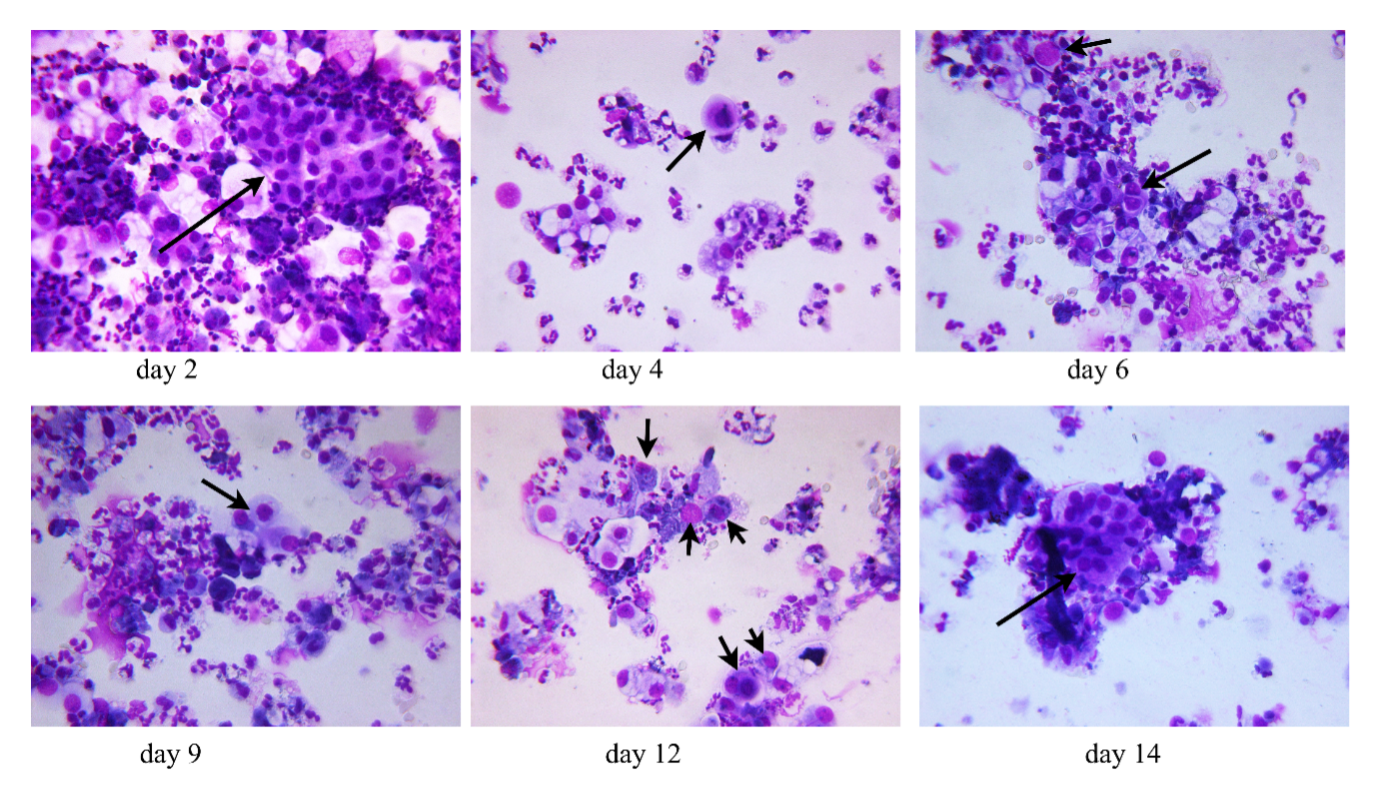

Supplement: Supplemental Information 1 — Seven to eight ml morning urine were centrifuged and the resultant pellet was resuspended in three ml tissue culture medium, 100µl were used to generate one cytopreparation. Following airdrying the slides were stained using routine H/E-staining. The arrow indicates hyperplastic epithelial clusters or cells with disproportionate cytoplasmic nuclear ratio. [file peerj-10-14110-s001.png]

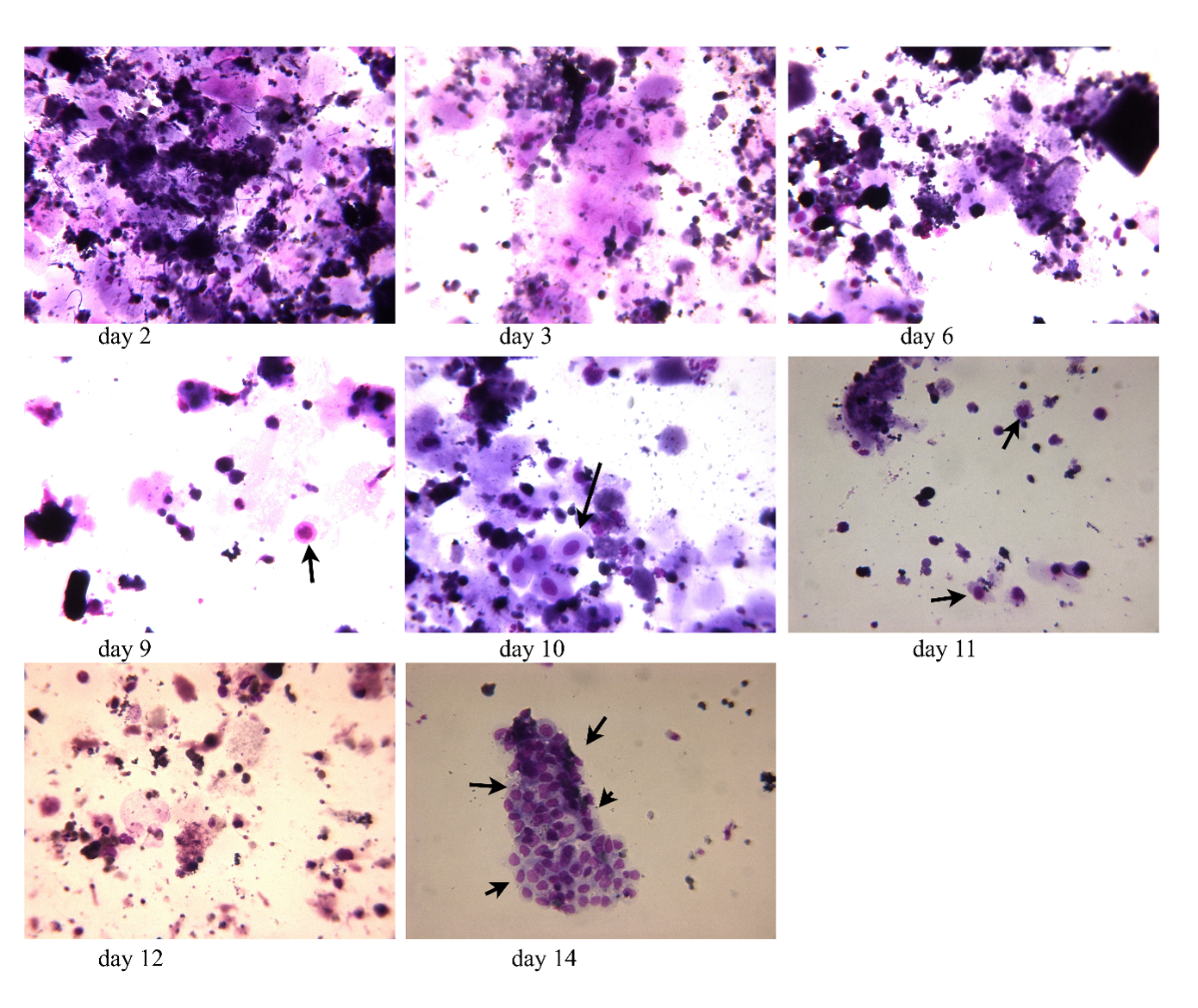

Supplement: Supplemental Information 2 — The arrow indicates hyperplastic epithelial clusters or cells with disproportionate cytoplasmic nuclear ratio. [file peerj-10-14110-s002.png]

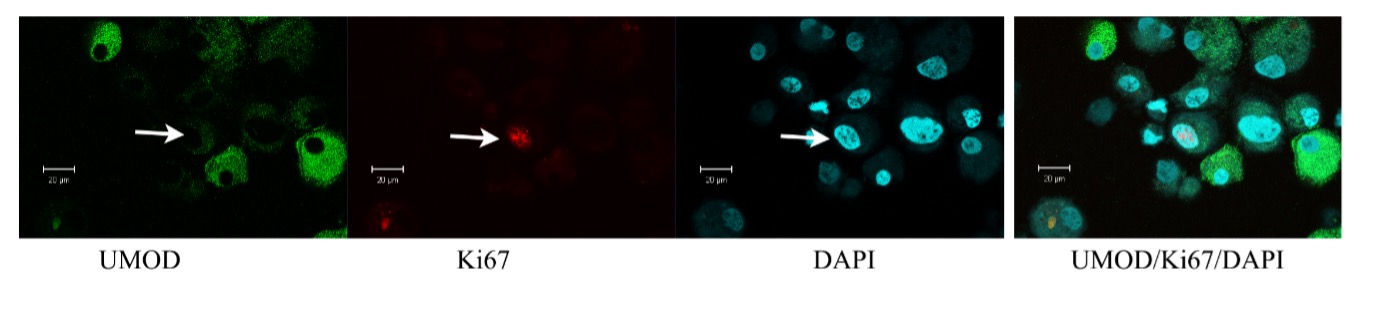

Supplement: Supplemental Information 3 — These maintained nuclear staining for Ki67 und showed UMOD staining to some degree. The nucleus is still disproportionate in size and directs towards the mitotic phase. [file peerj-10-14110-s003.jpg]

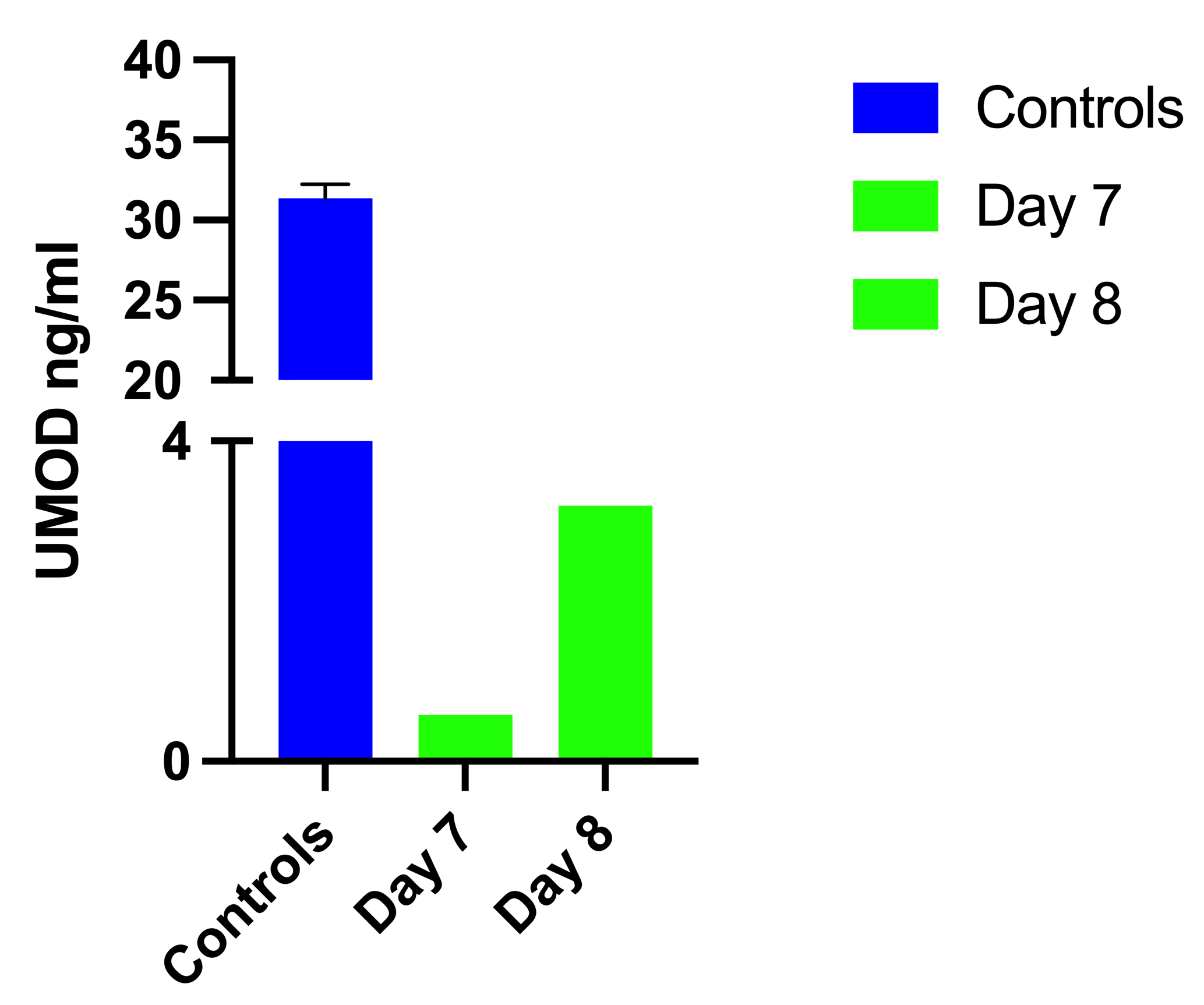

Supplement: Supplemental Information 4 — Three urine samples, diluted 1:10, were included as control (Controls). Tissue culture supernatant of tubular epithelial cells, which were kept in culture for 7 and 8 days were measured (Day 7, 8). The value represents 48h secretion per 10,000 cells. [file peerj-10-14110-s004.png]
